# Unveiling the influence of task-relevance of emotional faces on behavioral reactions in a multi-face context using a novel Flanker-Go/No-go task

**DOI:** 10.1038/s41598-023-47385-1

**Published:** 2023-11-17

**Authors:** Martina Montalti, Giovanni Mirabella

**Affiliations:** 1 Department of Clinical and Experimental Sciences, Brescia University, Viale Europa, 11, 25123 Brescia, Italy; 2 IRCCS Neuromed, Pozzilli, Italy

**Keywords:** Attention, Cognitive control, Human behaviour, Emotion

## Abstract

Recent research indicates that emotional faces affect motor control only when task-relevant. However, these studies utilized a single-face presentation, which does not accurately mirror real-life situations wherein we frequently engage with multiple individuals simultaneously. To overcome this limitation, we gave 40 participants two versions of a novel Flanker-Go/No-go task, where we presented three-face stimuli with a central target and two task-irrelevant flankers that could be congruent or incongruent with the target for valence and gender. In the Emotional Discrimination Task (EDT), participants had to respond to fearful or happy targets and refrain from moving with neutral ones. In the Gender Discrimination Task (GDT), the same images were shown, but participants had to respond according to the target's gender. In line with previous studies, we found an effect of valence only in EDT, where fearful targets increased reaction times and omission error rates compared to happy faces. Notably, the flanker effect, i.e., slower and less accurate responses in incongruent than congruent conditions, was not found. This likely stems from the higher perceptual complexity of faces than that of stimuli traditionally used in the Eriksen Flanker task (letters or signs), leading to a capacity limit in face feature processing.

## Introduction

The motivational theory^1,2^ states that emotions have a privileged processing pathway, being processed automatically. As a consequence, behavioral responses triggered by emotional stimuli, especially threatening ones, are postulated to be automatic and fixed, occurring independently from the context or individual's goals^3^. Nevertheless, the available empirical evidence does not align consistently with this hypothesis as the effects of valenced stimuli on behavioral reactions are extremely mixed and do not reveal a reproducible pattern^4–6^. The considerable heterogeneity of results can be accounted for by the fact that the impact of emotional information on behavior is not fixed. Rather, it is closely linked to the relevance of the stimuli in a specific context, as suggested by the appraisal theories of emotion^7,8^. For example, encountering a cobra in the wild may provoke a fight or flight reaction, whereas seeing the same snake hypnotized by a snake charmer can evoke interest and pleasure. In both situations, the observer may be close to the animal, but due to his/her knowledge and understanding, he/she appraises the stimulus differently, attributing it with distinct dangerousness and reacting accordingly. A recent series of studies by Mirabella and colleagues provide solid evidence in favor of appraisal theories of emotions by showing that emotional stimuli impact subjects’ behavioral performances only when task-relevant. The authors gave two versions of a Go/No-go task to the same group of healthy participants in a counterbalanced fashion (within-subject design). In the emotional version, participants were instructed to respond when an emotional stimulus was presented and to refrain from moving when a neutral stimulus appeared on the screen^4,6,9^, or vice versa^5,10^. By contrast, in the control task, identical images were presented, but participants were asked to respond according to aspects of the images’ features unrelated to their emotional valence, such as the posers’ gender or the color of their t-shirts. Regardless of the effectors employed [upper^5,6,9,10^, or lower limbs^4^], the stimuli’ emotions displayed [angry^4,6^, happy^4–6,9,10^, and fearful^5,6,9,10^ stimuli], and the type of motor control required [motor planning^4,6,9^, or motor inhibition^5,10^], all the evidence points to one conclusion: emotional stimuli influenced behavioral responses only when they were relevant to the task at hand. In such instances, threatening stimuli, i.e., angry and fearful expressions, capture and hold attention stronger than happy ones. However, when emotions were irrelevant to the task, the emotional connotation of the stimuli had no impact on the responses. Crucially, across all of these studies, the arousal’s impact, i.e., the other dimension of emotional stimuli besides valence, was always controlled. This ensured that any influence on response modulation by affective items could not be attributed to their arousal levels.

An important limitation of the above-cited studies is that the emotional stimuli, either faces or body postures, were shown one at a time, and this may not reflect real-life situations where we often interact with multiple individuals simultaneously who may convey the same or different emotional messages. To overcome this limit and create a more valid ecological context, we merged an Eriksen Flanker task design with our Go/No-go paradigm. In the Eriksen Flanker task^11^, a central target (e.g., a right arrow) is flanked by two or four non-target stimuli which can be congruent (e.g., right-pointing arrows) or incongruent (e.g., left-pointing arrows) with the response required by the target. Participants are required to respond quickly and accurately to the target while disregarding flankers. Usually, participants respond with greater speed and accuracy when the irrelevant information presented by the flankers is congruent with the information conveyed by the target. On the other hand, when the flankers are incongruent with the target, participants tend to be slower and less accurate in their responses. This is because they need to focus their attention on the relevant information while simultaneously suppressing the influence of distracting irrelevant information from the flankers. Thus, the Eriksen Flanker task is an excellent tool for studying attentional processes and cognitive control.

In our Flanker-Go/No-go design, we showed stimuli formed by three faces arranged in a horizontal array. The central target face was flanked by two task-irrelevant faces, which could be congruent or incongruent with the target face for valence and gender. In the Emotional Discrimination Task (EDT), participants had to make a reaching movement when fearful or happy target faces were presented and refrain from moving when a neutral target face appeared. Differently, in the Gender Discrimination Task (GDT), the same images were shown, but participants had to respond according to the target's gender, disregarding its emotional valence. Our aim was to assess whether the effect of task-relevance could be generalized using a behavioral paradigm requiring a much higher degree of attention and cognitive control, as participants have to filter out not only the irrelevant stimulus dimension of the central target (i.e., either the posers’ emotion or the gender) but also that of the flankers.

**Figure 1:**
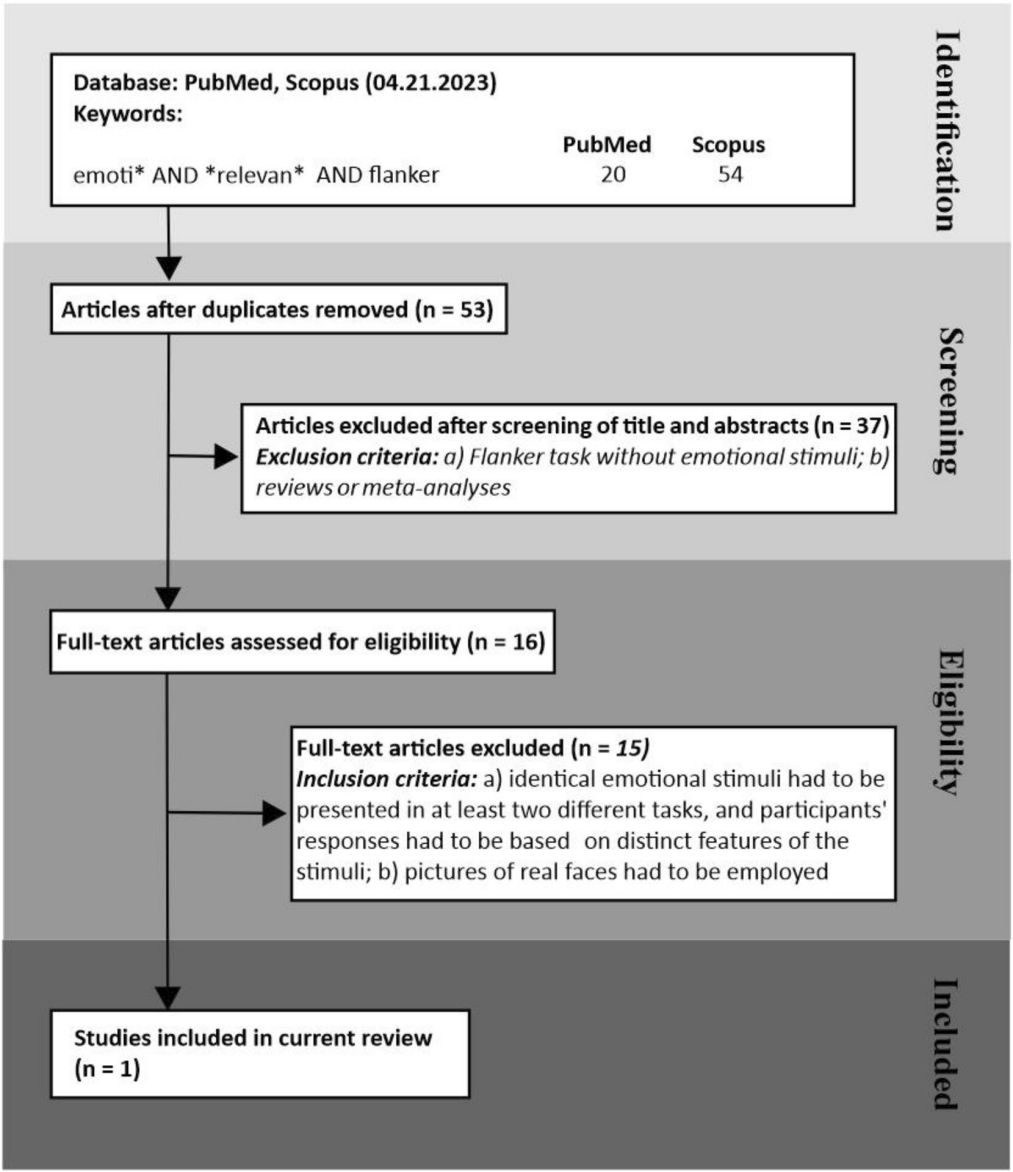
Flowchart of the search using PubMed and Scopus conducted on April 21, 2023. The keywords search was limited to titles and abstracts, but there was no limitation of publication dates or language. Fifty-three unique entries were found. We examined the full text for eligibility whenever an article could not be excluded based on its title or abstract. The inclusion criteria were that (a) the same emotional stimuli had to be presented in at least two different tasks, with participants responding to distinct features of the stimuli; (b) pictures of real faces had to be employed. One study satisfied such criteria.

To find whether previous studies tackled the issue of the task-relevance of emotions using an Eriksen flanker task, we conducted a systematic search in PubMed and Scopus, following the PRISMA guidelines see Fig. 1^12^. We identified 53 unique reports. Thirty-seven papers were excluded based on their title or abstract, while the full text of the remaining 13 were examined for eligibility. The inclusion criteria were that (1) identical emotional stimuli were presented in at least two different tasks, and participants had to respond to distinct features of the stimuli; (2) stimuli had to be pictures of real faces and not computer-generated or schematic faces. Only one article satisfied the criteria, Oldrati et al.^13^. To assess whether the processing of facial or body emotional expressions is automatic, they ran two studies. In the first one, they gave two versions of an affective Eriksen Flanker Task to 24 individuals. A horizontal array of three body postures or faces was presented, and participants were asked either to indicate the emotion or the gender of the central target by pressing a key while ignoring the flankers. Regardless of the type of stimuli and the task-relevance, the authors found that the emotional connotation of the stimuli impacted the performance. In fact, in both tasks, when the valence of central and flanker images matched, participants were faster and more accurate than when the valence was incongruent. By contrast, gender congruency/incongruency did not impact the performance. Thus, Oldrati et al.^13^ concluded that the emotional value of the stimuli could not be filtered out even when it was task-irrelevant. Yet, in the second experiment in which a new group of 24 participants was asked to determine whether the emotion or the gender of central and flanking stimuli matched, the researchers discovered that the emotional features impaired the same-different judgments only for bodies but not facial expressions. In other words, in the second experiment, the emotional connotation of faces could be filtered out, and the impact on behavior depended upon the stimuli task-relevance. These results are partially conflicting with our findings^4–6,9,10^. The most likely explanation could be that in each trial, we always presented a single stimulus, while Oldrati et al.^13^ presented an array of three pictures. Under these conditions, the attention control system might become unable to suppress emotionally irrelevant information from the flankers.

Our innovative experimental design is tailored to explore this possibility, thus providing crucial insights into whether the task-relevance effect of emotional stimuli persists even when the attentional and cognitive demands increase due to distracting information.

## Materials and methods

Prior to carrying out the study, we defined the sample size and inclusion/exclusion criteria, data rejection criteria, and statistical analysis to be used. The present section contains information on these methodological aspects.

### Participants

The sample size was estimated with G*Power 3.1.9.7^14^ for a repeated measures ANOVA and input variables (effect size f = 0.15, alpha = 0.05, power = 0.90, number of measures = 16, correlations among repeated measures r = 0.5, and non-sphericity correction e = 0.8) were estimated from previously published data^4,9^. The estimated minimum sample size was 39. Thus, we recruited 40 healthy volunteers (20 females; Mean ± Standard Deviation (M ± SD) age = 23.6 ± 4.1, range = 19.9–32.5). All participants were right-handed, as assessed by the Italian version of the Edinburgh handedness inventory^15^, and they had a normal or corrected-to-normal visual acuity. None of the participants had a history of psychiatric and neurological diseases and was informed about the purpose and the theoretical framework of the study. Before starting the study, participants provided written informed consent, approved by the local ethical committee “ASST Spedali Civili” di Brescia (protocol number 4452). All the procedures were conducted in accordance with the Declaration of Helsinki, 2013.

### Stimuli

**Figure 2:**
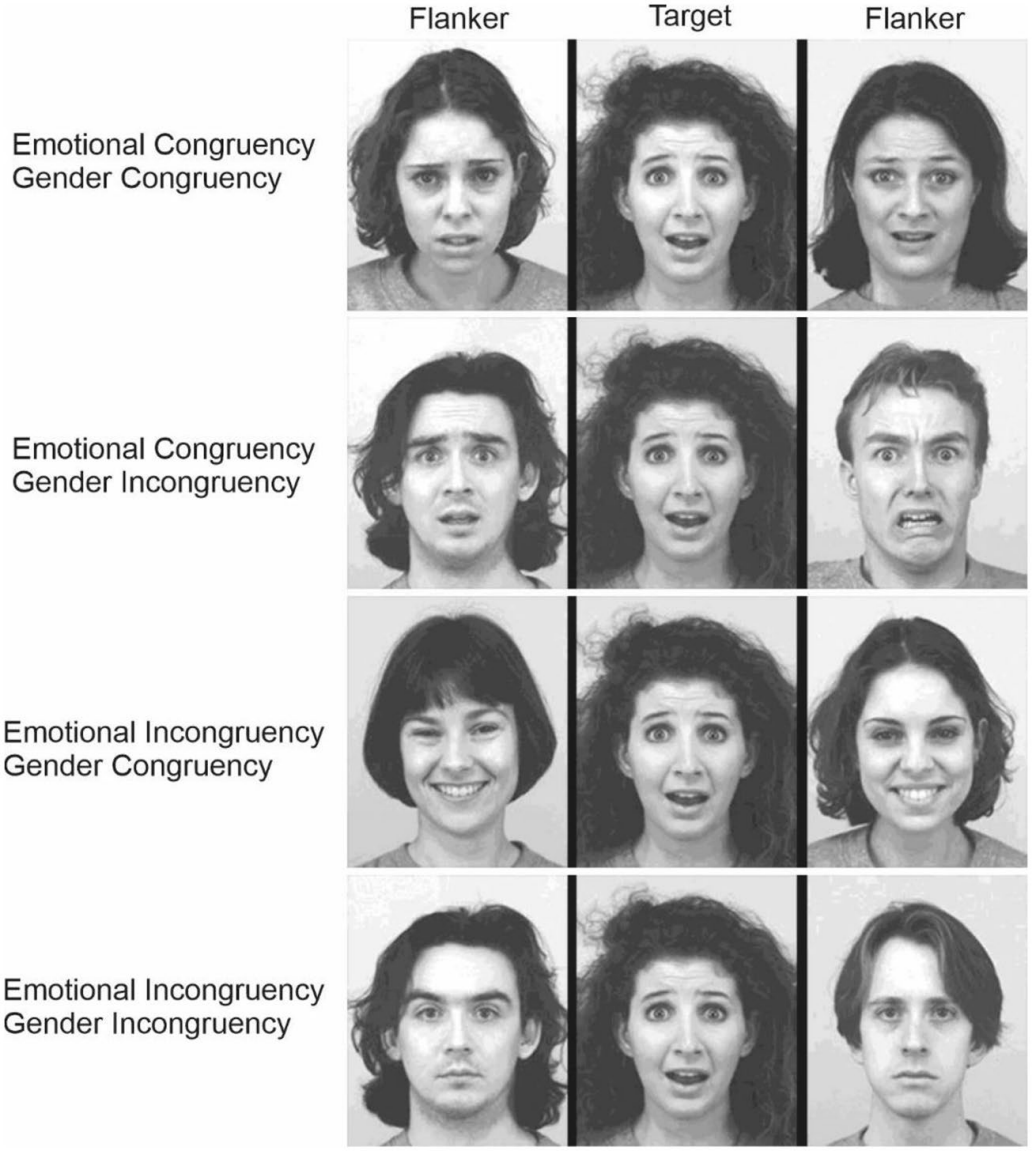
Experimental stimuli. 30 pictures of 10 different actors/actresses (half female) depicting fearful, happy, and neutral facial expressions were taken from the Karolinska Directed Emotional Faces (https://kdef.se/)^16^. Four individuals (two female) were used as target stimuli, while the remaining six individuals (three female) served as flankers. Both the target and flankers’ faces could depict fearful, happy, and neutral facial expressions. We balanced the emotional and gender characteristics according to the congruency or incongruency between target and flankers. In the picture, we show one example of the four possible combinations with a fearful female as the target.

The experimental stimuli were arrays with a row of three greyscale facial expressions taken from the Karolinska Directed Emotional Faces dataset (KDEF, https://kdef.se/)^16^. Pictures displayed faces of actors and actresses performing a happy, fearful, or neutral expression. The central stimulus was the target, while the sided stimuli were the flankers. As a target, we used four actors (two females), while as flankers we used six different actors (3 females). To ensure a balanced representation of emotions and genders, we controlled over the alignment or misalignment between the target and flankers. To illustrate, when our target was a happy female, we designed stimuli where she was flanked by either two female faces or two male faces. The flankers themselves could also align or misalign with the target in terms of emotional expression, meaning they could display happy, fearful, or neutral expressions. Notably, the flankers’ emotions and gender were always the same, i.e., we never had a condition in which one flanker was a happy face and the other was a fearful face, or one flanker was a male and the other one was a female. We ended up creating 72 distinct stimuli, which could be categorized into four categories: (a) stimuli that were congruent in terms of both emotion and gender, (b) stimuli that were congruent in terms of emotion but incongruent in terms of gender, (c) stimuli that were incongruent in terms of emotion but congruent in terms of gender, and (d) stimuli that were incongruent in terms of both emotion and gender (Fig. 2). It is important to note that in each single stimulus, the identities of the posers were always different to avoid pop-up effects.

At the end of the experimental session, participants were administered two separate questionnaires to assess the valence and arousal of the target face of each stimulus regardless of its flankers. Participants were asked to evaluate the arousal using a Likert scale that ranged from 1 (not at all arousing) to 7 (very much arousing). The valence was also evaluated on a Likert scale ranging from 1 (negative) through 4 (neutral) to 7 (positive). For each participant, we calculated the average rating of each target emotional dimension. Given that the Shapiro–Wilk tests revealed that not all valence/arousal ratings were normally distributed, we used a non-parametric Friedman rank-sum test to assess differences in targets' emotional ratings [within-participant factor: Emotion (3 levels: Fear, Happiness, and Neutral)]. As for valence ratings the analysis revealed a significant main effect of Emotion (χ^2^(2) = 78.05, *p* < 0.0001). As expected, post-hoc comparison with Bonferroni correction showed that happy facial expressions (M ± SD = 6.05 ± 0.69) were rated significantly more positive-valenced than both fearful (M ± SD = 2.14 ± 0.66) and neutral (M ± SD = 3.75 ± 0.39) ones. Moreover, fearful facial expressions were rated significantly more negative-valenced than neutral ones (all *p*_*s*_ < 0.0001). As for arousal ratings the analysis showed a significant main effect of Emotion (χ^2^(2) = 55.8, *p* < 0.0001). Post-hoc comparison with Bonferroni correction showed that both happy (M ± SD = 4.08 ± 0.95) and fearful (M ± SD = 4.66 ± 1.1) facial expressions were rated significantly more arousing than neutral ones (M ± SD = 2.03 ± 1.02, both *p*_*s*_ < 0.0001). Moreover, the analysis revealed that fearful expressions were rated more arousing than happy ones (*p* = 0.001).

### Experimental apparatus and procedure

**Figure 3:**
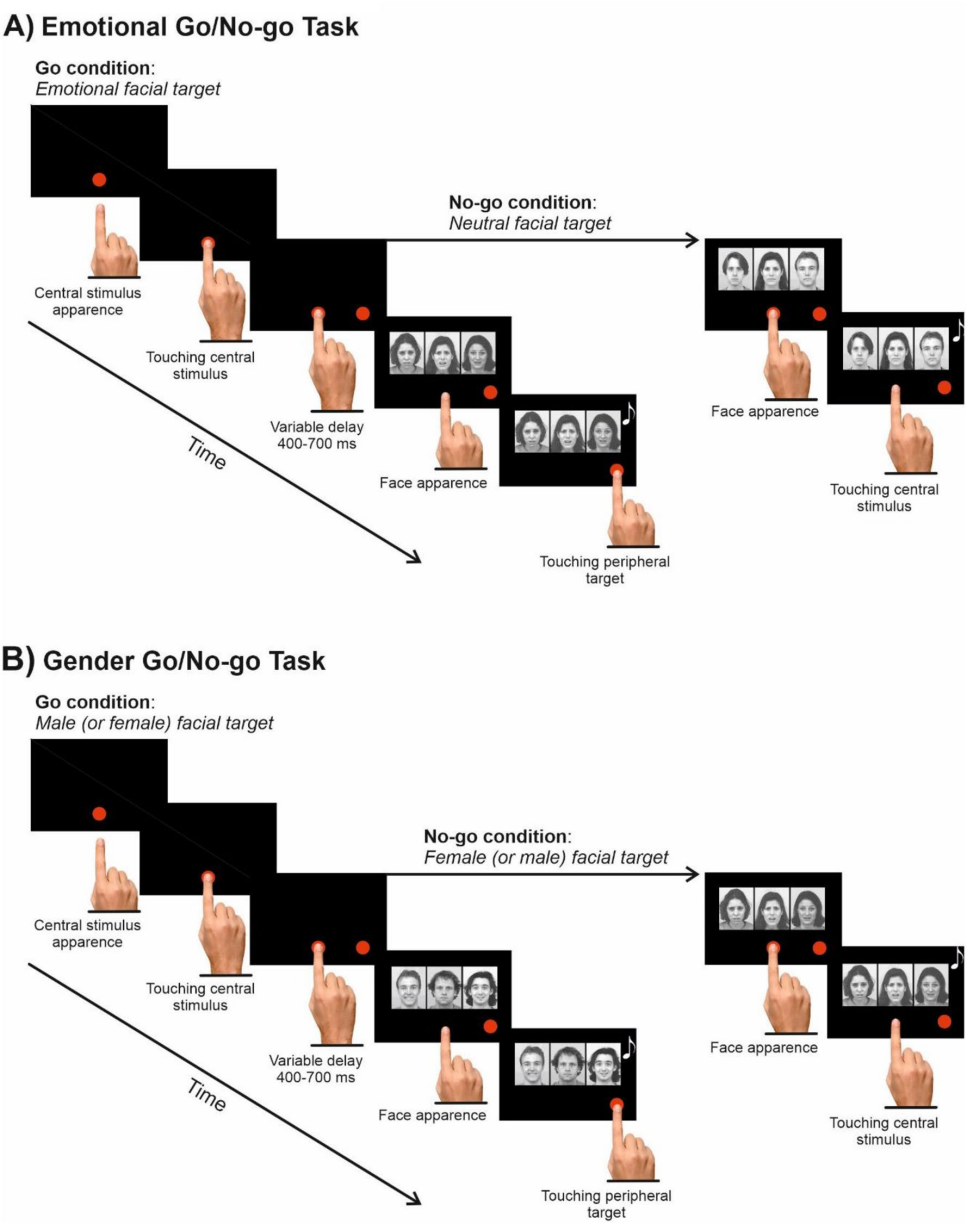
Experimental design. (**A**) In the Emotional Discrimination task, trials started with a red dot at the center of the screen. Participants were instructed to touch and hold the dot for a random delay of 400–700 ms. Then, a peripheral red dot appeared on the right side of the screen, followed by a stimulus constituted by three faces in a row. The central face, or the target, expressed a fearful, happy, or neutral expressions. The flankers on its side could depict also fear, happiness or neutral and could be congruent or incongruent with the target for either emotion or gender, or both. Participants were instructed to reach and hold the peripheral target when the face expressed an emotion (67%) and to refrain from moving if the face had a neutral expression (33%). (**B**) In the Gender Discrimination task, the trial structure was identical, but participants were instructed to respond or not according to the actor/actress’ gender. were taken from the Karolinska Directed Emotional Faces (https://kdef.se/)^16^.

In the same session, participants completed two different versions of the Flanker-Go/No-go task, the EDT and the GDT. The task order was counterbalanced across participants. In the EDT (Fig. 3A), each trial started with the presentation of a central red dot (2.43 cd/m^2^, diameter 2.8 cm or 4 dva) located 2 cm below the center of the screen. Participants were instructed to reach and touch the dot with the index finger of their dominant hand. As soon as the central dot was touched, a second one appeared on the right side of the screen (the target) at an eccentricity of 8 cm or 11.3 dva. Participants had to hold the central stimulus for a variable amount of time (400–700 ms) until it disappeared, while one of our three-faces-stimuli appeared just above the central dot (Go/No-go cue). Participants were instructed to reach as fast and accurately as possible the target and to hold it for a variable amount of time (300–400 ms) whenever the central face showed an emotion (go trials; 66%). By contrast, they had to refrain from moving when the central face was neutral (no-go trials; 34%), holding the central dot for a variable amount of time (400–800 ms). Acoustic feedback signaled correct trials. Participants had a maximum of 500 ms to respond to the target face (upper RT). We set this upper RTs limit to discourage participants from slowing down during the task. However, to avoid cutting the right tail of RTs distribution, we gave an extra 100 ms to respond to the target face. Therefore, when participants’ RTs were comprised between 500 and 600 ms, they were recorded, but the go-trials were signaled as errors and aborted (overtime reaching trials^17^). Overtime reaching-trials were included in the analyses, and they accounted for 4.57% of the total go-trials. The EDT had 576 trials, which were divided into three blocks to allow resting. The experimental conditions presentation was randomized and balanced across blocks.

The GDT (Fig. 3B) had the same stimuli and timing parameters as the EDT, but participants had to respond according to the gender of the central face. To avoid gender bias, half of the participants were instructed to move to the presentation of a female face and to refrain from moving when a male face was shown, and vice versa for the other half. Participants performed 576 trials (66% go and 34% no-go trials). Again, the experimental conditions presentation was randomized. To balance all the experimental conditions across blocks, we divided the task into four blocks to allow resting. The overtime reaching-trials were signaled as errors and included in the analyses. They accounted for 2.97% of the total go-trials.

Crucially, in the Emotional Discrimination task, we refrained from informing participants about the specific emotions they would encounter during the experiment. We instructed them to move at the presentation of facial emotion and to withhold the movement for neutral expression. Consequently, just as in the Gender Discrimination task, each behavior was mapped to one gender; similarly, in the Emotion Discrimination task, there was a one-to-one mapping between an emotional state and a behavioral response.

### Analyses

To investigate how emotions impact motor control, we considered three behavioral parameters: (i) RTs of correct go trials, i.e., the time between the go-signal appearance and the finger detach. Go trials whose RTs exceeded three standard deviations above and below the mean (EDT: 0.53%; GDT: 0.77%) were excluded; (ii) movement times of correct go trials, i.e., the time between the finger detach and the touch the peripheral dot; and (iii) the omission error rates percentage (OERs), i.e., instances in which participants did not move their index finger from the central dot although they had to. Separately for each participant and each condition, OERs were computed as the ratio between the number of omission errors and the overall number of trials multiplied by 100 (e.g., the OER of a fearful target flanked by neutral expressions in the EDT was calculated as the ratio between the number of omission errors and the overall number of go trials of the same condition).

The ratings of arousal differed for happy and fearful expressions; thus we included it as a factor in all the analyses. We used the Revised Standardized Difference Test (RSDT)^18^ to compare arousal ratings for happy and fearful faces. The RSDT enables the assessment of whether the standardized difference in ratings for individuals significantly deviates from the average difference observed in the remaining n-1 judgments, which serve as a control group. This method produces an index called Delta Arousal, indicating the rarity of an individual’s difference, expressed as a proportion relative to the population with a greater level of discrepancy. Using the Delta Arousal, we formed two subgroups of equal size. Participants whose standardized difference in arousal ratings fell below the 30th percentile or above the 70th percentile were categorized into the "High arousal" group, while those ranging between the 30th and 70th percentiles were included in the “Low arousal” group.

For each behavioral parameter, i.e., RTs, movement times, and ERs, we performed a five-way ANOVA with a mixed design [within-participants factors: Emotion (2 levels: fear, happiness); Task (2 levels: Emotion Discrimination task, Gender Discrimination task); Emotional congruency (i.e., whether the emotions of the flankers’ faces were or not congruent with the emotion expressed by the central face, 2 levels: congruent, incongruent); Gender congruency (i.e., whether the gender of the flankers’ faces were or not congruent with the gender of the central face, 2 levels: congruent, incongruent); between-participants factor: Delta Arousal (2 levels: high, low)]. Although not all variables were normally distributed as assessed by the Shapiro–Wilk tests (see Supplementary materials), we employed parametric tests as the size of our sample is large enough (> 30^19^) to make the parametric approach robust in deviations from normal distribution^20^. Furthermore, as in the go trials of the GDT, we presented fearful, happy, and neutral facial expressions; we evaluated differences in each behavioral parameter between such conditions via a series of parametric mixed design four-way ANOVA [within-participants factors: Emotion (3 levels: fear, happiness, neutral); Emotional congruency (2 levels: congruent, incongruent); Gender congruency (2 levels: congruent, incongruent); between-participants factor: Delta Arousal (2 levels: high, low)]. All post-hoc tests were perform using Bonferroni corrections. For each test, we reported the effect sizes as partial eta-squared (*η*^2^_*p*_) or Cohen’s d. Finally, Bayes Factors (BF_10_)^21^ were computed, setting the prior odds to 0.707 to quantify the null hypothesis’ strength (R package BayesFactor^22^). Values of BF_10_ > 3 and > 10 indicate moderate and strong support for the alternative hypothesis, respectively. Values of BF_10_ < 0.1 and < 0.33 indicate strong and substantial support for the null hypothesis; values 0.33 < BF_10_ < 3 are inconsistent for any hypothesis. All statistical analyses were performed using R, version 4.2.3 (R Core Team, 2023).

### Ethics statement

The study involves human participants. It was reviewed and approved by Local ethical committee “ASST Spedali Civili” of Brescia, Italy (protocol number 4452). Participants provided their written informed consent to participate in this study.

## Results

### Reaction times

**Figure 4:**
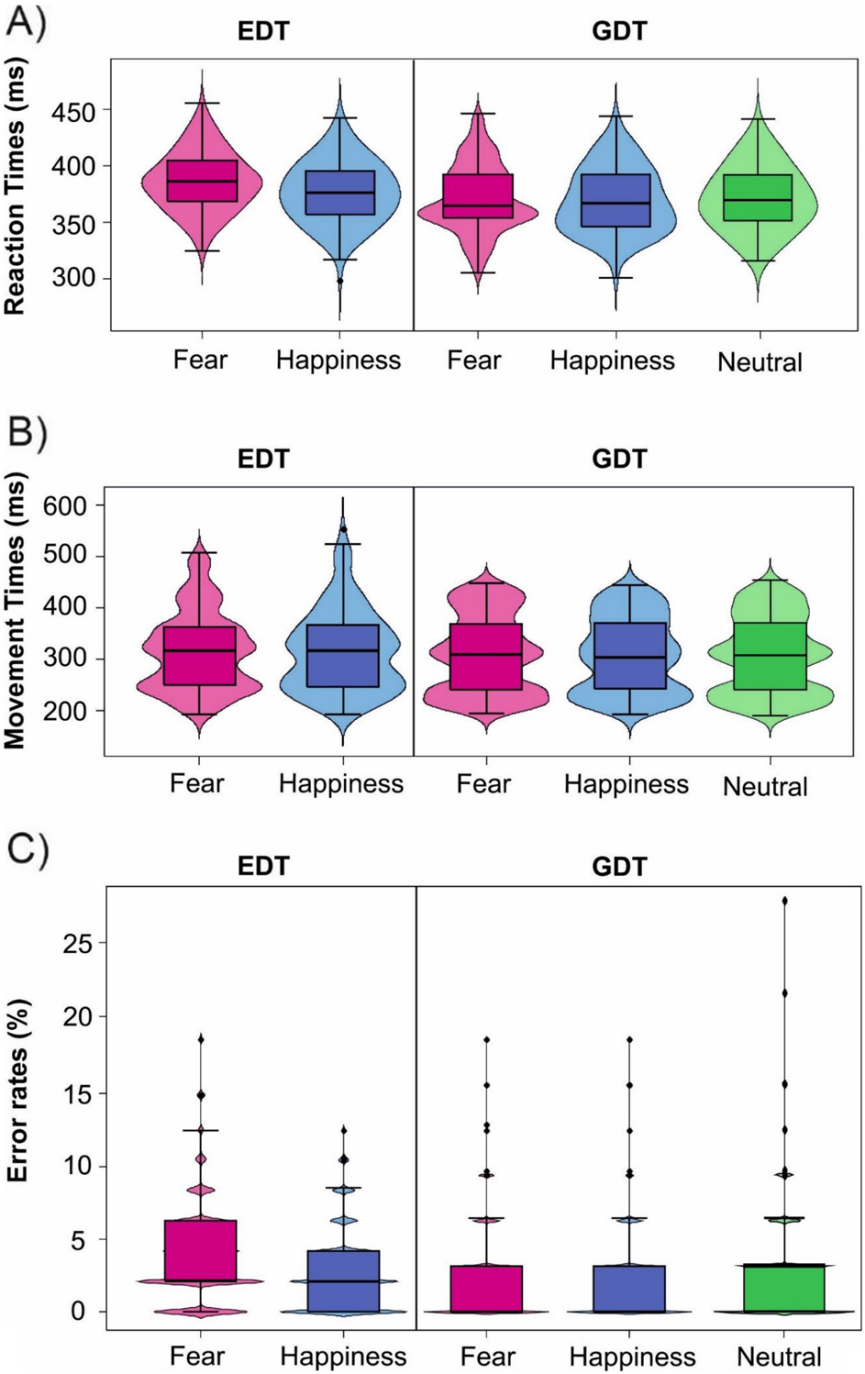
Effects of emotional facial expressions on behavioral parameters in the Emotional (EDT) and Gender Discrimination tasks (GDT). (**A**) Reaction times in the EDT were longer for fearful than for (i) happy facial expressions in the EDT and for (ii) fearful expressions in GDT. Conversely, no significant differences were found in the GDT. (**B**) Movement times. No differences were found in both tasks. (**C**) Percentage of omission errors (OER). The OER was higher for fearful than for happy expressions in the EDT, while in the GDT, there was no difference. For all graphs: boxplots are reported inside violin plots, which depict kernel probability density. The black line of the boxplot represents the median of the data, and the lower and the upper box’s boundaries indicate the first and third quartiles, respectively. The violin plot width shows the data frequency.

The five-way ANOVA on mean RTs (Table S1, i.e., in the Supplementary materials; Fig. 4A) of go trials revealed a main effect of Task (F(1,38) = 12.39, *p* = 0.001; *η*^2^_*p*_ = 0.25; BF_10_ > 100) because participants were faster in the GDT (M ± SD = 370.46 ± 29.41 ms) than the EDT (M ± SD = 381.49 ± 26.68 ms). It also revealed a main effect of Emotion (F(1,38) = 32.30; *p* < 0.001; *η*^2^_*p*_ = 0.46; BF_10_ > 100) since participants had longer RTs to respond to fearful facial expressions (M ± SD = 378.91 ± 28.88 ms) compared to the happy ones (M ± SD = 373.04 ± 28.05 ms). These main effects are qualified by a significant interaction effect between Task and Emotion (F(1,38) = 23.00; *p* < 0.001; *η*^2^_*p*_ = 0.38; BF_10_ = 35.92). In the EDT, fearful faces (M ± SD = 386.40 ± 25.80 ms) had higher RTs than happy ones (M ± SD = 376.59 ± 26.74 ms; *t*(38) = 6.89; *p* < 0.0001; *Cohen’s d* = 0.66; BF_10_ > 100). In contrast, emotional faces did not differ in the GDT (*t*(38) = 1.59; *p* = 0.241; *Cohen’s d* = 0.12; BF_10_ = 0.28).

### Movement times

The five-way ANOVA on mean movement times of go trials (Table S2; Fig. 4B) revealed only a significant interaction effect between Task and Emotional congruency (F(1,38) = 5.17; *p* = 0.029; *η*^2^_*p*_ = 0.12; BF_10_ = 0.18); however, no post-hoc comparison survives Bonferroni’s correction (all *p*_*s*_ > 0.05).

### Omission error rates

The five-way ANOVA on OERs (Table S3; Fig. 4C) showed a main effect of Emotion (F(1,38) = 6.12, *p* < 0.018; *η*^2^_*p*_ = 0.14; BF_10_ = 0.81) with a higher percentage of ERs in fearful (M ± SD = 3.18 ± 3.60) facial expressions than the happy ones (M ± SD = 2.68 ± 3.29). Moreover, the analysis showed a significant interaction effect between Task and Emotion (F(1,38) = 5.15; *p* < 0.05; *η*^2^_*p*_ = 0.12; BF_10_ = 1.28). Post hoc comparison revealed that in the EDT fearful facial expressions led to higher OERs (M ± SD = 3.79 ± 3.56) compared to the happy ones (M ± SD = 2.80 ± 2.97; *t*(38) = 2.84; *p* = 0.01; *Cohen’s d* = 0.27; BF_10_ = 20.52), while no difference was found in the GDT (*t*(38) = − 0.01; *p* = 1.00; *Cohen’s d* = − 0.00; BF_10_ = 0.09).

Relevantly, in none of the previous analyses, we found either a main effect or an interaction concerning emotion/gender congruency or arousal.

The ANOVA analyses on the GDT, comparing the RTs, movement times, and the OERs of fearful, happy, and neutral facial expressions, did not show any significant effect (all *p*_*s*_ > 0.05).

## Discussion

In this study, we used a novel task design, the Flanker-Go/No-go task, to investigate the role of task-relevance of emotional stimuli in triggering behavioral reactions under heightened attentional demands. To respond correctly, participants, on the one hand, had to focus their attention on the central target disregarding the flankers. Still, on the other hand, they also have to attend to the relevant target’s feature, i.e., either the emotional expression or the gender. Thus, both spatial- and feature-based attention were simultaneously required in our task. Our results were straightforward and consistent with previous research. Participants were capable of filtering out both the flanking stimuli and the irrelevant target dimension. This suggests that the attentional control system can suppress all information void of relevance to produce the correct response. Thus, as in past studies^4–6,9,10^, we found that the impact of emotional stimuli on behavioral reactions is not automatic but conditional to the relevance of the emotional dimension to the top–down instructions of the ongoing task. Again, fearful expressions impaired performance, resulting in longer RTs and lower accuracy than happy expressions in the EDT but not in the GDT^4,6,9^. Of great importance, all our results are net of the arousal dimension and depend solely on stimuli valence and cannot be ascribed to the perceptual characteristics of stimuli because we showed the same images in the two tasks. Finally, all significant findings exhibit substantial effect sizes and are supported by high values of BF_10_, providing robust statistical evidence in favor of the alternative hypothesis. Conversely, all crucial non-significant results are accompanied by values of BF_10_, suggesting a higher likelihood of the null hypothesis. Therefore, our evidence is statistically very solid.

### The impact of task-relevance in the Eriksen flanker task

Our results indicate that the features of the two unattended flanking faces never intruded in the main task, i.e., we never observed the classical Eriksen flankers effect^11^, i.e., participants respond slower and less accurately in the incongruent condition compared to the congruent condition. Thus, at least under our experimental conditions a space-based filtering mechanisms successfully canceled the conflict created by incongruent flankers. Such evidence is in contrast with the results of Oldrati et al.^13^, who, in their affective version of the Flanker task, found that the emotional dimension of stimuli affected participants’ performance when either relevant (emotion task) or irrelevant (gender task) for the task at hand. Several methodological differences can explain the discrepancy. First, Oldrati et al.^13^ had a smaller sample size (24 vs. 40 participants), fewer trial (192 vs. 576 trials for each task) and did not assess the arousal of the stimuli, leaving the possibility that arousal, instead of valence could determine the observed effect. Interestingly, however, Oldrati et al.^13^ in another task, where participants had to report whether the emotion or the gender of the central target and the flanking stimuli matched, found that facial emotional features could be filtered out.

Interestingly, Zhou and Liu^23^, using a paradigm very similar to Oldrati et al.^13^ found somewhat different results. Their findings revealed that the flanker effect manifested solely in the dimension pertinent to the task. Specifically, it emerged for emotions in the emotional task and for gender in the gender task, thus suggesting that top–down mechanisms would allow suppressing the influence of the flankers’ task-irrelevant feature but not the ones of the task-relevant feature. Differently from Oldrati et al.^13^ and from us, Zhou and Liu^23^ used computer-generated faces and they used a single face identity. However, in our opinion, the main difference between our study and the previous two is that we never required participants to provide a classification of emotions or gender. Instead, we instructed people to use visual information for acting not for perceiving, and it is widely recognized that the visual signals employed in shaping our perceptions differ from those governing our motor actions^24^.

### The lack of the classical Eriksen Flanker effect

**Figure 5:**
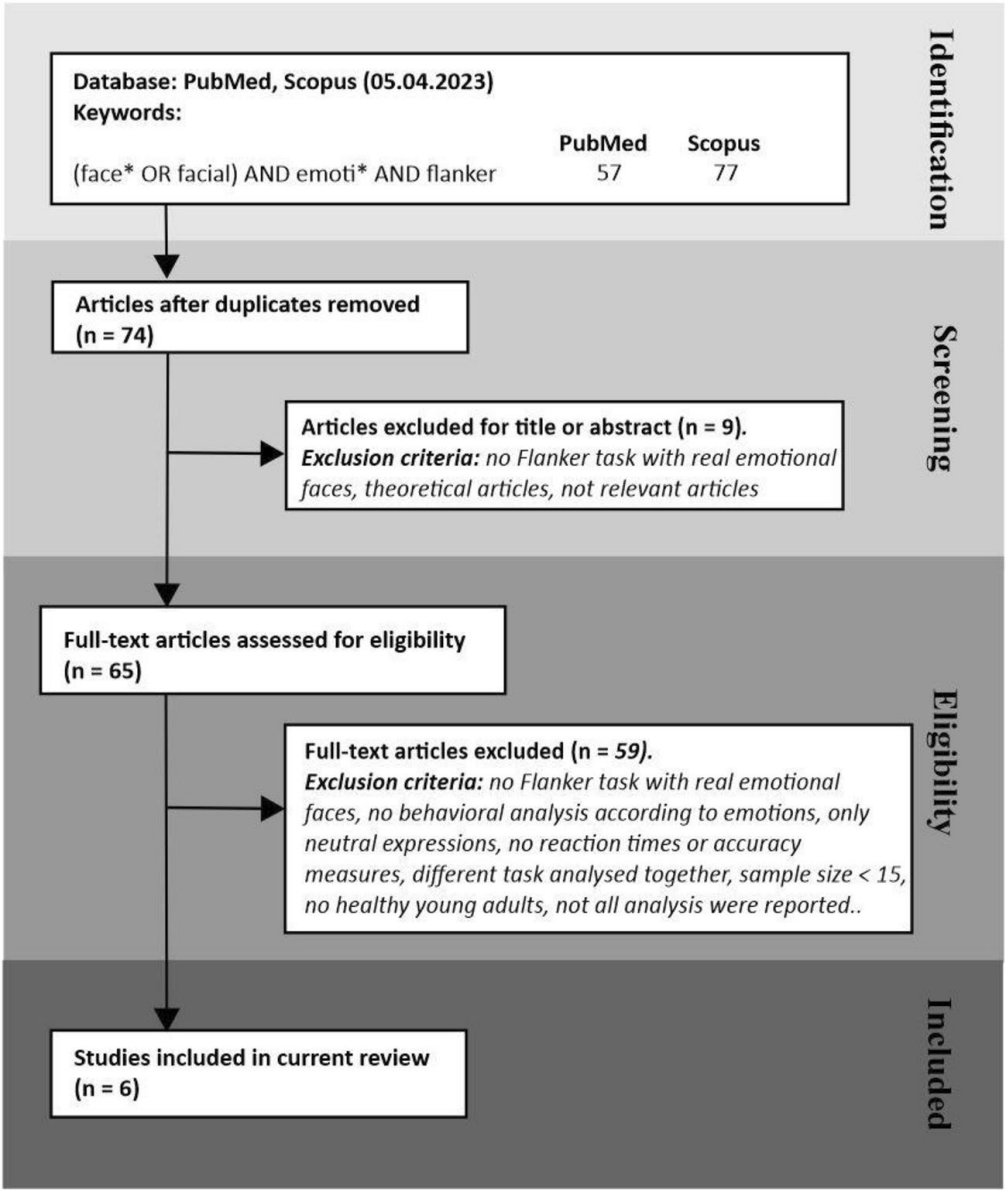
Flowchart of the search using PubMed and Scopus conducted on May 5, 2023. The keywords search was limited to titles and abstracts, but there was no limitation of publication dates or language. Seventy-four unique entries were found. Nine were eliminated after screening titles and abstracts as they referred to different populations (e.g., children, older adults) or studies employing computer-generated or schematic faces. We examined the full text of the remaining articles following the exclusion criteria indicated in the corresponding box, and we included six studies.

Given that our study did not show the classical flanker effect, i.e., participants did not respond faster and with greater accuracy in congruent than incongruent conditions, while Oldrati et al.^13^ and Zhou and Liu^23^ gave discrepant results, we conducted a literature search in Pubmed and Scopus databases following the PRISMA guidelines see Fig. 5^12^, to investigate the effect of congruency in experiments where flankers were employed in healthy young adults. We identified 74 unique reports. Nine papers were excluded based on their title or abstract, while the full text of the remaining 65 was examined for eligibility. We excluded studies (a) using schematic, morphed, or computer-generated faces as stimuli, (b) employing task-irrelevant emotional faces as priming stimuli before displaying the conventional Eriksen Flanker stimuli, (c) incorporating task-irrelevant emotional faces as background stimuli, over which the traditional Eriksen Flanker stimuli were superimposed, (d) that did not perform behavioral analyses according to congruence/incongruence of target *vs*. flankers’ emotions. Six articles satisfied the criteria. In all cases (Table 1), emotions were task-relevant as the instruction was either to discriminate the emotional expression of the target stimulus^25–29^ or to press/refrain from pressing a key according to the facial emotion of the central target^30^.

**Table 1:** Eriksen Flanker task studies employing emotional faces resulted from the searches.

	Sample size	Task	Trials	Stimuli	Results: accuracy	Results: RTs
Ashley and Swick, 2019	23	**Affective Eriksen flanker task** Emotional categorization Stimuli: array of 1 × 3 faces (identical flankers)	432	***Emotions***: A, F, N ***Databases:*** ADFES, KDEF, WSEFEP, RaFD	Target: **N > F and A** Congruency: Ø Target*Congruency: A congruent > A incongruent	Target: **N < F < A** Congruency: Ø Target*Congruency: Ø
Boncompagni and Casagrande, 2019	134	**Affective Eriksen flanker task** Emotional categorization Stimuli: array of 1 × 5 faces (identical flankers, the identity of the target was the same as the flankers)	N/A	***Emotions:*** THREAT, N ***Database:*** Not validated	Target: **N > THREAT** Congruency: Ø Target*Congruency: N congruent > N incongruent	Target: **THREAT < N** Congruency: Congruent < Incongruent Target*Congruency: N congruent < N incongruent
du Rocher and Pickering, 2023	87	**Affective Eriksen flanker task** Emotional categorization Stimuli: 3 × 3 array (identical flankers)	240	***Emotions:*** F, H ***Database:*** NimStim	Target: **H > F** Congruency: Ø Target*Congruency: Ø	Target: **H < F** Congruency: Ø Target*Congruency: Ø
Liu et al., 2018	28	**Affective Flanker-Go/No-go task** Responses were related to one emotion Stimuli: 1X5 array (identical flankers, the identity of the target was the same as the flankers)	960	***Emotions***: F, H ***Database*:** Not validated	N/A^a^	Target: **H < F** Congruency: Ø Target*Congruency: Ø
Moser et al., 2008	21	**Affective Eriksen flanker task** Emotional categorization Stimuli: array of 1 × 5 faces (identical flankers, the identity of the target was the same as the flankers)	576	***Emotions***: THREAT (A, D); REASS (H, Su) ***Database*:** Not validated	Target: Ø Congruency: Ø Target*Congruency: Ø	Target: **REASS < THREAT** Congruency: Congruent < Incongruent Target*Congruency: Ø
Mueller and Kuchinke, 2016	40	**Affective Eriksen flanker task** Emotional categorization Stimuli: array of 1 × 5 faces (identical flankers, the identity of the target was the same as the flankers)	400	***Emotions***: A, H ***Database:*** RaFD	Target: Ø Congruency: Congruent > Incongruent Target*Congruency: Ø	Target: Ø Congruency: Congruent < Incongruent Target*Congruency: Ø

*ADFES* Amsterdam Dynamic Facial Expression set, *KDEF* Karolinska Directed Emotional Faces, *WSEFEP* Warsaw Set of Emotional Facial Expression Pictures, *RaFD* Radboud Faces Database, *NimStim* Set of Facial Expressions, *H* Happiness, *F* Fear, *A* Anger, *Su* Surprise, *D* Disgust, *N* Neutral, *THREAT* Threatening, *REASS* Reassuring, *Ø* no significant effect, *N/A* Not available. ^a^Authors analysed the rate of omission errors in Go trials and commission errors in No-go trials together, i.e. they used a faulty statistical approach. Thus, we did not report results.

Results were highly inconsistent. In three cases out of six, a congruency effect in terms of RTs was not found^25,27,30^. In the other three studies, the effect was as expected^26,28,29^. However, Boncompagni and Casagrande^26^ found that the effect was limited to neutral expressions. As far as accuracy is concerned, in two cases out of five one study was excluded because of a faulty statistical analysis^30^, there was no effect of congruency^25,28^. In two studies, the effect of congruency was limited to only one facial expression, i.e., angry^27^ or neutral faces^26^. Finally, Mueller and Kuchinke^29^ found an overall higher accuracy in the congruent than incongruent condition. Thus, only one research found a full flanker effect^29^. Methodological differences are likely to explain the differences between these studies, e.g., the lack of a response time window, which eliminates time-related urgency, the number and the choice of flankers, and the lack of arousal assessment, which leaves open the possibility that this factor might be responsible for the observed effect. This crucial dimension of emotional stimuli was assessed only twice^28,30^. In both cases, the stimuli arousal did not differ, but in one case, the congruency effect was absent^30^, whereas in the other, it was only evident in RTs.

From our perspective, although some caveats must be kept in mind, the existing body of literature supports the idea that the congruency effect is not observed when emotional faces are employed as flankers. The lack of the flanker effect aligns with the notion that faces have a capacity limit for visual processing so that no more than one face can be processed at a time^31–33^ and this applies not only to cognitively complex processes such as the extraction of personal identity^34^, but also to cognitively more basic processes such as categorizing gender^32^. Recently, Qarooni et al.^35^ found that we can detect more than one face at a time, and thus, they suggested that coarse face/non-face discriminations may be conducted in parallel before further face information is extracted. Instead, finer discriminations among faces (e.g., judgements about gender or eye direction) are performed in series because processing one face precludes processing another. Taken together, these findings indicate that the heightened perceptual complexity of faces, the consequent capacity limits in face perception coupled with the instructions of paying attention only to the central faces can enable participants to effectively exclude the surrounding flanker faces, thus minimizing conflict. Differently, the conventional version of the Eriksen Flanker task involves using perceptually simple stimuli, such as letters or < > signs. In this scenario, cognitive resources are sufficient for processing simultaneously the central target and the flankers, consequently giving rise to conflict under incongruent conditions.

### The impact of positive and negative emotional target faces on behavioral responses

Our literature search highlighted another relevant piece of evidence by showing that (a) in five out of six cases (83%), RTs were longer when the target face had a threatening expression (fearful, angry or disgusted) than for positive (happy and surprise) or neutral faces and (b) in three out of five cases (60%) the accuracy was higher for threatening than positive or neutral faces. This evidence perfectly aligns with our current and past findings^4,6,9^, and indicates that when emotions are task-relevant, negative emotions hold attention more strongly than positive or neutral expressions.

## Conclusions

The present study confirmed the role of task-relevance of emotional stimuli on behavioral reactions shown in previous works^4,5,9,10,36^ even under heightened attentional demands. Presenting more faces at the same time made the experimental design more ecologically valid and indicates that participants can focus on the relevant faces fully ignoring the flanking ones. As such, our findings can contribute to another hotly debated issue, i.e., whether or not emotional faces can be more easily detected in a crowd. At present, literature does not bring to a unique conclusion as some researchers sustain that happy facial expressions can be detected more efficiently^37^, whereas others sustain that angry faces pop-out of crowds^38^. We suggest that inconsistencies might derive primarily from the relevance of the emotional stimulus.

Our conclusions clash with the existence of motivational patterns that automatically drive our behavior in response to emotional stimuli, particularly threatening ones^1–3^. Conversely, our findings support the appraisal theories of emotions suggesting that emotional responses can be adjusted in accordance with the current context’s demands^7,8,39,40^.

### Supplementary Information

Supplementary Tables.

## Data Availability

The datasets presented in this study can be found in online repositories. The names of the repository/repositories and accession number(s) can be found at: https://osf.io/j6cvs/.
