# Supplementary material for: Unveiling the influence of task-relevance of emotional faces on behavioral reactions in a multi-face context using a novel Flanker-Go/No-go task
Source: Sci Rep. 2023 Nov 17;13:20183. doi: 10.1038/s41598-023-47385-1 (PMC10656465; doi:10.1038/s41598-023-47385-1)
Supplement: Supplementary file 1 — Supplementary Tables. [file 41598_2023_47385_MOESM1_ESM.docx]

**Unveiling the Influence of Task-Relevance of Emotional Faces on Behavioral Reactions in a Multi-Face Context Using a Novel Flanker-Go/No-Go Task**

**Supplementary material**

**Table S1**

*Statistical analysis results of go trials reaction times (RTs).*

| **Five-way parametric ANOVA on RTs Within-Participant Factors: Emotion (2 levels: fear, happiness); Task (2 levels: Emotion Discrimination task, Gender Discrimination task); Emotional congruency (2 levels: congruent, incongruent); Gender congruency (2 levels: congruent, incongruent); Between-Participant Factor: Delta Arousal (2 levels: high, low)** | | | | | | | | | | | | |  |  |
| --- | --- | --- | --- | --- | --- | --- | --- | --- | --- | --- | --- | --- | --- | --- |
| **Effect** |  | **Value of parameters** | | ***p-*values** | **M_diff_** | | **95% CI** | | **Effect Size** | | **BF_10_** | | | |
| Main | Delta Arousal | | F(1,38) = 0.26 | .612 | | - | | - | | η²ₚ = 0.007 | | 0.51 | |  |
|  | **Task** | | **F(1,38) = 12.39** | **.001** | | **11.03** | | **[8.36; 13.71]** | | **η²ₚ = 0.246** | | **> 100** | |  |
|  | **Emotion** | | **F(1,38) = 32.30** | **< .001** | | **5.86** | | **[4.13; 7.59]** | | **η²ₚ = 0.459** | | **> 100** | |  |
|  | Emotional congruency | | F(1,38) = 3.84 | .058 | | - | | - | | η²ₚ = 0.092 | | 0.25 | |  |
|  | Gender congruency | | F(1,38) = 0.30 | .586 | | - | | - | | η²ₚ = 0.008 | | 0.09 | |  |
| Two-way interaction | Delta Arousal * Task | | F(1,38) = 0.06 | .804 | | - | | - | | η²ₚ = 0.002 | | 0.16 | |  |
|  | Delta Arousal * Emotion | | F(1,38) = 0.05 | .816 | | - | | - | | η²ₚ = 0.001 | | 0.11 | |  |
|  | Delta Arousal * Emotional congruency | | F(1,38) = 0.03 | .868 | | - | | - | | η²ₚ < 0.001 | | 0.11 | |  |
|  | Delta Arousal * Gender congruency | | F(1,38) = 0.36 | .550 | | - | | - | | η²ₚ = 0.009 | | 0.14 | |  |
|  | **Task * Emotion** | | **F(1,38) = 23.00** | **< .001** | | **-** | | **-** | | **η²ₚ = 0.377** | | **35.92** | |  |
|  | **Post-hoc comparisons of the interaction effect Task * Emotion** | | | | | | | | | | | | |  |
|  | **Emotional Discrimination task:**  **Fear *vs.* Happiness** | | **t(38) = 6.89** | **< .001** | | **9.81** | | **[7.50; 12.11]** | | **D = 0.66** | | **> 100** | |  |
|  | Gender Discrimination task:  Fear *vs.* Happiness | | t(38) = 1.59 | .241 | | 1.92 | | [-0.53; 4.37] | | D = 0.12 | | 0.28 | |  |
|  | Task * Emotional congruency | | F(1,38) = 0.03 | .874 | | - | | - | | η²ₚ < 0.001 | | 0.12 | |  |
|  | Task * Gender congruency | | F(1,38) = 0.42 | .520 | | - | | - | | η²ₚ = 0.011 | | 0.14 | |  |
|  | Emotion * Emotional congruency | | F(1,38) = 0.49 | .487 | | - | | - | | η²ₚ = 0.013 | | 0.15 | |  |
|  | Emotion * Gender congruency | | F(1,38) = 0.00 | .978 | | - | | - | | η²ₚ < 0.001 | | 0.13 | |  |
|  | Emotional congruency * Gender congruency | | F(1,38) = 3.67 | .063 | | - | | - | | η²ₚ = 0.088 | | 0.32 | |  |
| Three-way interaction | Delta Arousal * Task * Emotion | | F(1,38) = 0.49 | .490 | | - | | - | | η²ₚ = 0.013 | | 0.22 | |  |
|  | Delta Arousal * Task * Emotional congruency | | F(1,38) = 0.74 | .394 | | - | | - | | η²ₚ = 0.019 | | 0.11 | |  |
|  | Delta Arousal * Task * Gender congruency | | F(1,38) = 1.99 | .167 | | - | | - | | η²ₚ = 0.050 | | 0.44 | |  |
|  | Delta Arousal * Emotion * Emotional congruency | | F(1,38) = 0.07 | .799 | | - | | - | | η²ₚ = 0.002 | | 0.19 | |  |
|  | Delta Arousal * Emotion * Gender congruency | | F(1,38) = 0.43 | .518 | | - | | - | | η²ₚ = 0.011 | | 0.22 | |  |
|  | Delta Arousal * Emotional congruency * Gender congruency | | F(1,38) = 0.35 | .556 | | - | | - | | η²ₚ = 0.009 | | 0.15 | |  |
|  | Task * Emotion * Emotional congruency | | F(1,38) = 0.94 | .337 | | - | | - | | η²ₚ = 0.024 | | 0.24 | |  |
|  | Task * Emotion * Gender congruency | | F(1,38) = 2.18 | .148 | | - | | - | | η²ₚ = 0.054 | | 0.22 | |  |
|  | Task * Emotional congruency * Gender congruency | | F(1,38) = 2.12 | .154 | | - | | - | | η²ₚ = 0.053 | | 0.34 | |  |
|  | Emotion * Emotional congruency * Gender congruency | | F(1,38) = 0.19 | .662 | | - | | - | | η²ₚ = 0.005 | | 0.14 | |  |
| Four-way interaction | Delta Arousal * Task * Emotion * Emotional congruency | | F(1,38) = 0.36 | .554 | | - | | - | | η²ₚ = 0.009 | | 0.32 | |  |
|  | Delta Arousal * Task * Emotion * Gender congruency | | F(1,38) = 0.45 | .505 | | - | | - | | η²ₚ = 0.012 | | 0.22 | |  |
|  | Delta Arousal * Task * Emotional congruency * Gender congruency | | F(1,38) = 0.21 | .651 | | - | | - | | η²ₚ = 0.005 | | 0.14 | |  |
|  | Delta Arousal * Emotion * Emotional congruency * Gender congruency | | F(1,38) = 0.00 | .978 | | - | | - | | η²ₚ < 0.001 | | 0.20 | |  |
|  | Task * Emotion * Emotional congruency * Gender congruency | | F(1,38) = 0.48 | .493 | | - | | - | | η²ₚ = 0.012 | | 0.22 | |  |
| Five-way interaction | Delta Arousal * Task * Emotion * Emotional congruency * Gender congruency | | F(1,38) = 0.06 | .809 | | - | | - | | η²ₚ = 0.002 | | 0.03 | |  |
|  |  | |  |  | |  | |  | |  | |  | |  |
| **Four-way parametric ANOVA on RTs Within-Participants Factors: Emotion (3 levels: fear, happiness, neutral); emotional congruency (2 levels: congruent, incongruent); gender congruency (2 levels: congruent, incongruent); Between-Participant Factor: Delta Arousal (2 levels: high, low)** | | | | | | | | | | | | | |  |
| **Effect** |  | | **Value of parameters** | ***p-*values** | | **M_diff_** | | **95% CI** | | **Effect Size** | | **BF_10_** | |  |
| Main | Delta Arousal | | F(1,38) = 0.06 | .801 | | - | | - | | η²ₚ = 0.002 | | 0.48 | |  |
|  | Emotion | | F(1.93, 73.15) = 1.85 | .166 | | - | | - | | η²ₚ = 0.046 | | 0.12 | |  |
|  | Emotional congruency | | F(1,38) = 0.50 | .484 | | - | | - | | η²ₚ = 0.013 | | 0.14 | |  |
|  | Gender congruency | | F(1,38) = 0.01 | .913 | | - | | - | | η²ₚ < 0.001 | | 0.11 | |  |
| Two-way interaction | Delta Arousal * Emotion | | F(1.93, 73.15) = 0.91 | .405 | | - | | - | | η²ₚ = 0.023 | | 0.10 | |  |
|  | Delta Arousal * Emotional congruency | | F(1,38) = 0.03 | .855 | | - | | - | | η²ₚ < 0.001 | | 0.14 | |  |
|  | Delta Arousal * Gender congruency | | F(1,38) = 0.90 | .348 | | - | | - | | η²ₚ = 0.023 | | 0.23 | |  |
|  | Emotion * Emotional congruency | | F(1.98, 75.37) = 1.17 | .316 | | - | | - | | η²ₚ = 0.030 | | 0.10 | |  |
|  | Emotion * Gender congruency | | F(1.93, 73.51) = 0.52 | .593 | | - | | - | | η²ₚ = 0.013 | | 0.09 | |  |
|  | Emotional congruency * Gender congruency | | F(1,38) = 0.09 | .765 | | - | | - | | η²ₚ = 0.002 | | 0.15 | |  |
| Three-way interaction | Delta Arousal * Emotion * Emotional congruency | | F(1.98, 75.37) = 0.09 | .916 | | - | | - | | η²ₚ = 0.002 | | 0.11 | |  |
|  | Delta Arousal * Emotion * Gender congruency | | F(1.93, 73.51) = 0.38 | .678 | | - | | - | | η²ₚ = 0.010 | | 0.07 | |  |
|  | Delta Arousal * Emotional congruency * Gender congruency | | F(1,38) = 0.11 | .745 | | - | | - | | η²ₚ = 0.003 | | 0.21 | |  |
|  | Emotion * Emotional congruency * Gender congruency | | F(1.93, 73.46) = 0.02 | .975 | | - | | - | | η²ₚ < 0.001 | | 0.06 | |  |
| Four-way interaction | Delta Arousal * Emotion * Emotional congruency * Gender congruency | | F(1.93, 73.46)= 0.22 | .798 | | - | | - | | η²ₚ = 0.006 | | 0.57 | |  |

*Delta Arousal = index of the arousal difference between fearful and happy expressions (see text for more details); Effect size = partial eta squared (η²ₚ) for the ANOVAs and Cohen’s d for the post-hoc tests; BF_10_ = Bayes Factors report the ratio of likelihood of the alternative hypothesis to the likelihood of the null hypothesis; p-values were reported in bold when < 0.05; alpha level in post-hoc (i.e., pairwise) comparisons were adjusted according to Bonferroni correction.* *Differences in the estimated marginal means (Mdiff) and their 95% confidence interval (CI) are reported.*

**Table S2**

*Statistical analysis results of go trials movement times (MTs).*

| **Five-way parametric ANOVA on MTs Within-Participant Factors: Emotion (2 levels: fear, happiness); Task (2 levels: Emotion Discrimination task, Gender Discrimination task); Emotional congruency (2 levels: congruent, incongruent); Gender congruency (2 levels: congruent, incongruent); Between-Participant Factor: Delta Arousal (2 levels: high, low)** | | | | | | | | | | | | |  |  |
| --- | --- | --- | --- | --- | --- | --- | --- | --- | --- | --- | --- | --- | --- | --- |
| **Effect** |  | **Value of parameters** | | ***p-*values** | **M_diff_** | | **95% CI** | | **Effect Size** | | **BF_10_** | | | |
| Main | Delta Arousal | | F(1,38) = 0.01 | .916 | | - | | - | | η²ₚ < 0.001 | | 0.68 | |  |
|  | Task | | F(1,38) = 1.60 | .213 | | - | | - | | η²ₚ = 0.040 | | **>100** | |  |
|  | Emotion | | F(1,38) = 0.04 | .852 | | - | | - | | η²ₚ < 0.001 | | **0.08** | |  |
|  | Emotional congruency | | F(1,38) = 0.51 | .482 | | - | | - | | η²ₚ = 0.013 | | 0.09 | |  |
|  | Gender congruency | | F(1,38) = 0.07 | .800 | | - | | - | | η²ₚ = 0.002 | | 0.09 | |  |
| Two-way interaction | Delta Arousal * Task | | F(1,38) = 0.30 | .588 | | - | | - | | η²ₚ = 0.008 | | 1.04 | |  |
|  | Delta Arousal * Emotion | | F(1,38) = 0.00 | .995 | | - | | - | | η²ₚ < 0.001 | | 0.13 | |  |
|  | Delta Arousal * Emotional congruency | | F(1,38) = 0.01 | .926 | | - | | - | | η²ₚ < 0.001 | | 0.12 | |  |
|  | Delta Arousal * Gender congruency | | F(1,38) = 1.82 | .185 | | - | | - | | η²ₚ = 0.046 | | 0.13 | |  |
|  | Task * Emotion | | F(1,38) = 0.20 | .657 | | - | | - | | η²ₚ = 0.005 | | **0.14** | |  |
|  | **Task * Emotional congruency** | | **F(1,38) = 5.17** | **.029** | | **-** | | **-** | | η²ₚ = **0.120** | | **0.18** | |  |
|  | **Post-hoc comparisons of the interaction effect Task * Emotional congruency** | | | | | | | | | | | | |  |
|  | Emotional Discrimination task:  Emotional congruency vs. incongruency | | t(38) = -2.502 | .067 | | -1.72 | | [-3.44; -0.007] | | -0.157 | | 0.16 | |  |
|  | Gender Discrimination task:  Emotional congruency vs. incongruency | | t(38) = 0.999 | 1.00 | | 0.92 | | [-0.69; 2.52] | | 0.089 | | 0.59 | |  |
|  | Emotional congruency:  Emotional Discrimination task vs Gender Discrimination task | | t(38) = 1.025 | 1.00 | | 6.12 | | [0.15; 12.09] | | 0.160 | | 0.64 | |  |
|  | Emotional incongruency:  Emotional Discrimination task vs Gender Discrimination task | | t(38) = 1.500 | .567 | | 8.76 | | [2.85; 14.67] | | 0.231 | | 5.32 | |  |
|  | Task * Gender congruency | | F(1,38) = 0.55 | .464 | | - | | - | | η²ₚ = 0.014 | | 0.11 | |  |
|  | Emotion * Emotional congruency | | F(1,38) = 0.79 | .381 | | - | | - | | η²ₚ = 0.020 | | 0.15 | |  |
|  | Emotion * Gender congruency | | F(1,38) = 0.46 | .502 | | - | | - | | η²ₚ = 0.012 | | 0.11 | |  |
|  | Emotional congruency * Gender congruency | | F(1,38) = 0.10 | .752 | | - | | - | | η²ₚ = 0.003 | | 0.11 | |  |
| Three-way interaction | Delta Arousal * Task * Emotion | | F(1,38) = 0.01 | *.905* | | - | | - | | η²ₚ < 0.001 | | 0.11 | |  |
|  | Delta Arousal * Task * Emotional congruency | | F(1,38) = 0.00 | *.960* | | - | | - | | η²ₚ < 0.001 | | 0.16 | |  |
|  | Delta Arousal * Task * Gender congruency | | F(1,38) = 0.00 | *.958* | | - | | - | | η²ₚ < 0.001 | | 0.31 | |  |
|  | Delta Arousal * Emotion * Emotional congruency | | F(1,38) = 0.49 | *.486* | | - | | - | | η²ₚ = 0.013 | | 0.25 | |  |
|  | Delta Arousal * Emotion * Gender congruency | | F(1,38) = 1.70 | *.200* | | - | | - | | η²ₚ = 0.043 | | 0.15 | |  |
|  | Delta Arousal * Emotional congruency * Gender congruency | | F(1,38) = 0.00 | *>.999* | | - | | - | | η²ₚ < 0.001 | | 0.23 | |  |
|  | Task * Emotion * Emotional congruency | | F(1,38) = 2.14 | .152 | | - | | - | | η²ₚ = 0.053 | | 0.23 | |  |
|  | Task * Emotion * Gender congruency | | F(1,38) = 0.29 | .591 | | - | | - | | η²ₚ = 0.008 | | 0.12 | |  |
|  | Task * Emotional congruency * Gender congruency | | F(1,38) = 0.16 | .692 | | - | | - | | η²ₚ = 0.004 | | 0.15 | |  |
|  | Emotion * Emotional congruency * Gender congruency | | F(1,38) = 0.01 | .938 | | - | | - | | η²ₚ < 0.001 | | 0.15 | |  |
| Four-way interaction | Delta Arousal * Task * Emotion * Emotional congruency | | F(1,38) = 0.80 | *.378* | | - | | - | | η²ₚ = 0.021 | | 0.15 | |  |
|  | Delta Arousal * Task * Emotion * Gender congruency | | F(1,38) = 1.12 | *.296* | | - | | - | | η²ₚ = 0.029 | | 0.10 | |  |
|  | Delta Arousal * Task * Emotional congruency * Gender congruency | | F(1,38) = 0.06 | *.813* | | - | | - | | η²ₚ = 0.001 | | 0.05 | |  |
|  | Delta Arousal * Emotion * Emotional congruency * Gender congruency | | F(1,38) = 0.03 | *.859* | | - | | - | | η²ₚ < 0.001 | | 0.11 | |  |
|  | Task * Emotion * Emotional congruency * Gender congruency | | F(1,38) = 0.49 | *.490* | | - | | - | | η²ₚ = 0.013 | | 0.16 | |  |
| Five-way interaction | Delta Arousal * Task * Emotion * Emotional congruency * Gender congruency | | F(1,38) = 0.65 | .426 | | - | | - | | η²ₚ = 0.017 | | 2.54 | |  |
|  |  | |  |  | |  | |  | |  | |  | |  |
| **Four-way parametric ANOVA on MTs Within-Participants Factors: Emotion (3 levels: fear, happiness, neutral); emotional congruency (2 levels: congruent, incongruent); gender congruency (2 levels: congruent, incongruent); Between-Participant Factor: Delta Arousal (2 levels: high, low)** | | | | | | | | | | | | | |  |
| **Effect** |  | | **Value of parameters** | ***p-*values** | | **M_diff_** | | **95% CI** | | **Effect Size** | | **BF_10_** | |  |
| Main | Delta Arousal | | F(1,38) = 0.00 | .982 | | - | | - | | η²ₚ < 0.001 | | 0.85 | |  |
|  | Emotion | | F(2, 75.84) = 0.22 | .803 | | - | | - | | η²ₚ = 0.006 | | 0.03 | |  |
|  | Emotional congruency | | F(1,38) = 0.07 | .794 | | - | | - | | η²ₚ = 0.002 | | 0.10 | |  |
|  | Gender congruency | | F(1,38) = 0.24 | .624 | | - | | - | | η²ₚ = 0.006 | | 0.11 | |  |
| Two-way interaction | Delta Arousal * Emotion | | F(2, 75.84) = 0.04 | .960 | | - | | - | | η²ₚ < 0.001 | | 0.06 | |  |
|  | Delta Arousal * Emotional congruency | | F(1,38) = 0.00 | .994 | | - | | - | | η²ₚ < 0.001 | | 0.11 | |  |
|  | Delta Arousal * Gender congruency | | F(1,38) = 1.58 | .214 | | - | | - | | η²ₚ = 0.040 | | 0.25 | |  |
|  | Emotion * Emotional congruency | | F(1.79, 68.09) = 0.90 | .400 | | - | | - | | η²ₚ = 0.023 | | 0.14 | |  |
|  | Emotion * Gender congruency | | F(1.87, 71.07) = 0.46 | .621 | | - | | - | | η²ₚ = 0.012 | | 0.08 | |  |
|  | Emotional congruency * Gender congruency | | F(1,38) = 0.40 | .533 | | - | | - | | η²ₚ = 0.010 | | 0.17 | |  |
| Three-way interaction | Delta Arousal * Emotion * Emotional congruency | | F(1.79, 68.09) = 0.50 | .589 | | - | | - | | η²ₚ = 0.013 | | 0.80 | |  |
|  | Delta Arousal * Emotion * Gender congruency | | F(1.87, 71.07) = 1.10 | .334 | | - | | - | | η²ₚ = 0.028 | | 0.12 | |  |
|  | Delta Arousal * Emotional congruency * Gender congruency | | F(1,38) = 0.44 | .510 | | - | | - | | η²ₚ = 0.012 | | 0.41 | |  |
|  | Emotion * Emotional congruency * Gender congruency | | F(1.84, 69.80) = 0.09 | .896 | | - | | - | | η²ₚ =.002 | | 0.04 | |  |
| Four-way interaction | Delta Arousal * Emotion * Emotional congruency * Gender congruency | | F(1.84, 69.80) = 0.29 | .727 | | - | | - | | η²ₚ = 0.008 | | 0.10 | |  |

*Delta Arousal = index of the arousal difference between fearful and happy expressions (see text for more details); Effect size = partial eta squared (η²ₚ) for the ANOVAs and Cohen’s d for the post-hoc tests; BF_10_ = Bayes Factors report the ratio of likelihood of the alternative hypothesis to the likelihood of the null hypothesis; p-values were reported in bold when < 0.05; alpha level in post-hoc (i.e., pairwise) comparisons were adjusted according to Bonferroni correction. Differences in the estimated marginal means (Mdiff) and their 95% confidence interval (CI) are reported.*

**Table S3**

*Statistical analysis results of go trials percentage of omission errors (OERs).*

| **Five-way parametric ANOVA on OERs Within-Participant Factors: Emotion (2 levels: fear, happiness); Task (2 levels: Emotion Discrimination task, Gender Discrimination task); Emotional congruency (2 levels: congruent, incongruent); Gender congruency (2 levels: congruent, incongruent); Between-Participant Factor: Delta Arousal (2 levels: high, low)** | | | | | | | | | | | | |  |  |
| --- | --- | --- | --- | --- | --- | --- | --- | --- | --- | --- | --- | --- | --- | --- |
| **Effect** |  | **Value of parameters** | | ***p-*values** | **M_diff_** | | **95% CI** | | **Effect Size** | | **BF_10_** | | | |
| Main | Delta Arousal | | F(1,38) = 3.73 | .061 | | - | | - | | η²ₚ = 0.089 | | 0.98 | |  |
|  | **Task** | | **F(1,38) = 3.11** | **.086** | | **0.73** | | **[0.25; 1.20]** | | **η²ₚ = 0.076** | | **11.84** | |  |
|  | **Emotion** | | **F(1,38) = 6.12** | **.018** | | **0.50** | | **[0.09; 0.91]** | | **η²ₚ = 0.139** | | **0.81** | |  |
|  | Emotional congruency | | F(1,38) = 1.90 | .177 | | - | | - | | η²ₚ = 0.048 | | 0.27 | |  |
|  | Gender congruency | | F(1,38) = 0.09 | .762 | | - | | - | | η²ₚ = 0.002 | | 0.09 | |  |
| Two-way interaction | Delta Arousal * Task | | F(1,38) = 0.20 | .655 | | - | | - | | η²ₚ = 0.005 | | 0.17 | |  |
|  | Delta Arousal * Emotion | | F(1,38) = 0.26 | .616 | | - | | - | | η²ₚ = 0.007 | | 0.13 | |  |
|  | Delta Arousal * Emotional congruency | | F(1,38) = 1.31 | .259 | | - | | - | | η²ₚ = 0.033 | | 0.25 | |  |
|  | Delta Arousal * Gender congruency | | F(1,38) = 0.17 | .679 | | - | | - | | η²ₚ = 0.005 | | 0.14 | |  |
|  | **Task * Emotion** | | **F(1,38) = 5.15** | **.029** | | **-** | | **-** | | **η²ₚ = 0.119** | | **1.28** | |  |
|  | **Post-hoc comparisons of the interaction effect Task * Emotion** | | | | | | | | | | | | |  |
|  | **Emotional Discrimination task:**  **Fear *vs.* Happiness** | | **t(38) = 2.84** | **.014** | | **1.00** | | **[0.41; 1.58]** | | ***d* = 0.268** | | **20.52** | |  |
|  | Gender Discrimination task:  Fear *vs.* Happiness | | t(38) = -.01 | 1.00 | | 0.00 | | [-0.57; 0.57] | | *d* = -0.00 | | 0.09 | |  |
|  | Task * Emotional congruency | | F(1,38) = 0.00 | .973 | | - | | - | | η²ₚ < 0.001 | | 0.12 | |  |
|  | Task * Gender congruency | | F(1,38) = 0.97 | .331 | | - | | - | | η²ₚ = 0.025 | | 0.17 | |  |
|  | Emotion * Emotional congruency | | F(1,38) = 0.02 | .889 | | - | | - | | η²ₚ < 0.001 | | 0.13 | |  |
|  | Emotion * Gender congruency | | F(1,38) = 0.75 | .392 | | - | | - | | η²ₚ = 0.019 | | 0.17 | |  |
|  | Emotional congruency * Gender congruency | | F(1,38) = 0.09 | .766 | | - | | - | | η²ₚ = 0.002 | | 0.12 | |  |
| Three-way interaction | Delta Arousal * Task * Emotion | | F(1,38) = 0.45 | .504 | | - | | - | | η²ₚ = 0.012 | | 0.23 | |  |
|  | Delta Arousal * Task * Emotional congruency | | F(1,38) = 0.69 | .410 | | - | | - | | η²ₚ = 0.018 | | 0.29 | |  |
|  | Delta Arousal * Task * Gender congruency | | F(1,38) = 0.04 | .843 | | - | | - | | η²ₚ = 0.001 | | 0.19 | |  |
|  | Delta Arousal * Emotion * Emotional congruency | | F(1,38) = 1.93 | .173 | | - | | - | | η²ₚ = 0.048 | | 0.44 | |  |
|  | Delta Arousal * Emotion * Gender congruency | | F(1,38) = 2.71 | .108 | | - | | - | | η²ₚ = 0.067 | | 0.55 | |  |
|  | Delta Arousal * Emotional congruency * Gender congruency | | F(1,38) = 0.49 | .488 | | - | | - | | η²ₚ = 0.013 | | 0.16 | |  |
|  | Task * Emotion * Emotional congruency | | F(1,38) = 1.14 | .292 | | - | | - | | η²ₚ = 0.029 | | 0.26 | |  |
|  | Task * Emotion * Gender congruency | | F(1,38) = 0.23 | .637 | | - | | - | | η²ₚ = 0.006 | | 0.17 | |  |
|  | Task * Emotional congruency * Gender congruency | | F(1,38) = 1.17 | .286 | | - | | - | | η²ₚ = 0.030 | | 0.30 | |  |
|  | Emotion * Emotional congruency * Gender congruency | | F(1,38) = 1.14 | .293 | | - | | - | | η²ₚ = 0.029 | | 0.23 | |  |
| Four-way interaction | Delta Arousal * Task * Emotion * Emotional congruency | | F(1,38) = 0.00 | .966 | | - | | - | | η²ₚ < 0.001 | | 0.13 | |  |
|  | Delta Arousal * Task * Emotion * Gender congruency | | F(1,38) = 0.26 | .611 | | - | | - | | η²ₚ = 0.007 | | 0.31 | |  |
|  | Delta Arousal * Task * Emotional congruency * Gender congruency | | F(1,38) = 0.34 | .561 | | - | | - | | η²ₚ = 0.009 | | 0.16 | |  |
|  | Delta Arousal * Emotion * Emotional congruency * Gender congruency | | F(1,38) = 0.00 | .998 | | - | | - | | η²ₚ < 0.001 | | 0.20 | |  |
|  | Task * Emotion * Emotional congruency * Gender congruency | | F(1,38) = 0.90 | .349 | | - | | - | | η²ₚ = 0.023 | | 0.18 | |  |
| Five-way interaction | Delta Arousal * Task * Emotion * Emotional congruency * Gender congruency | | F(1,38) = 0.65 | .425 | | - | | - | | η²ₚ =.017 | | 0.34 | |  |
|  |  | |  |  | |  | |  | |  | |  | |  |
| **Four-way parametric ANOVA on OERs Within-Participants Factors: Emotion (3 levels: fear, happiness, neutral); emotional congruency (2 levels: congruent, incongruent); gender congruency (2 levels: congruent, incongruent); Between-Participant Factor: Delta Arousal (2 levels: high, low)** | | | | | | | | | | | | | |  |
| **Effect** |  | | **Value of parameters** | ***p-*values** | | **M_diff_** | | **95% CI** | | **Effect Size** | | **BF_10_** | |  |
| Main | Delta Arousal | | F(1,38) = 2.53 | .120 | | - | | - | | η²ₚ = 0.063 | | 0.80 | |  |
|  | Emotion | | F(1,38) = 1.63 | .207 | | - | | - | | η²ₚ = 0.041 | | 0.11 | |  |
|  | Emotional congruency | | F(1,38) = 3.68 | .063 | | - | | - | | η²ₚ = 0.088 | | 0.50 | |  |
|  | Gender congruency | | F(1,38) = 0.68 | .413 | | - | | - | | η²ₚ = 0.018 | | 0.12 | |  |
| Two-way interaction | Delta Arousal * Emotion | | F(1,38) = 0.40 | .629 | | - | | - | | η²ₚ = 0.010 | | 0.06 | |  |
|  | Delta Arousal * Emotional congruency | | F(1,38) = 1.36 | .250 | | - | | - | | η²ₚ = 0.035 | | 0.23 | |  |
|  | Delta Arousal * Gender congruency | | F(1,38) = 0.09 | .768 | | - | | - | | η²ₚ = 0.002 | | 0.15 | |  |
|  | Emotion * Emotional congruency | | F(1,38) = 0.61 | .539 | | - | | - | | η²ₚ = 0.016 | | 0.08 | |  |
|  | Emotion * Gender congruency | | F(1,38) = 0.13 | .876 | | - | | - | | η²ₚ = 0.003 | | 0.05 | |  |
|  | Emotional congruency * Gender congruency | | F(1,38) = 0.00 | .995 | | - | | - | | η²ₚ < 0.001 | | 0.15 | |  |
| Three-way interaction | Delta Arousal * Emotion * Emotional congruency | | F(1,38) = 0.90 | .407 | | - | | - | | η²ₚ = 0.023 | | 0.15 | |  |
|  | Delta Arousal * Emotion * Gender congruency | | F(1,38) = 0.88 | .416 | | - | | - | | η²ₚ = 0.023 | | 0.20 | |  |
|  | Delta Arousal * Emotional congruency * Gender congruency | | F(1,38) = 0.01 | .921 | | - | | - | | η²ₚ < 0.001 | | 0.19 | |  |
|  | Emotion * Emotional congruency * Gender congruency | | F(1,38) = 1.21 | .300 | | - | | - | | η²ₚ = 0.031 | | 0.24 | |  |
| Four-way interaction | Delta Arousal * Emotion * Emotional congruency * Gender congruency | | F(1,38) = 0.96 | .377 | | - | | - | | η²ₚ = 0.025 | | 0.24 | |  |

*Delta Arousal = index of the arousal difference between fearful and happy expressions (see text for more details); Effect size = partial eta squared (η²ₚ) for the ANOVAs and Cohen’s d for the post-hoc tests; BF_10_ = Bayes Factors report the ratio of likelihood of the alternative hypothesis to the likelihood of the null hypothesis; p-values were reported in bold when < 0.05; alpha level in post-hoc (i.e., pairwise) comparisons were adjusted according to Bonferroni correction. Differences in the estimated marginal means (Mdiff) and their 95% confidence interval (CI) are reported.*
